# Examining the effect of neighborhood sanitation coverage on childhood diarrheal disease in rural Bangladesh

**DOI:** 10.1016/j.ijheh.2025.114732

**Published:** 2025-12-09

**Authors:** Hannah Van Wyk, Andrew F. Brouwer, Jesse Contreras, Mahbubur Rahman, Mahfuza Islam, Amy J. Pickering, Benjamin F. Arnold, Stephen P. Luby, John M. Colford, Matthew Freeman, Ayse Ercumen, Joseph N.S. Eisenberg

**Affiliations:** 1 Department of Epidemiology, University of Michigan School of Public Health, Ann Arbor, Michigan, USA; 2 Environmental Health and WASH, Health Systems and Population Studies Division, International Centre for Diarrhoeal Disease Research Bangladesh (icddr,b), Dhaka 1212, Bangladesh; 3 Global Health and Migration United, Department of Women’s and Children’s Health, Uppsala University, Sweden; 4 Department of Environmental Health Sciences, School of Public Health, University of California, Berkeley, Berkeley, CA 94720, USA; 5 Department of Civil and Environmental Engineering, University of California, Berkeley, Berkeley, CA 94720, USA; 6 Chan Zuckerberg Biohub, San Francisco, CA 94158, USA; 7 Francis I. Proctor Foundation, University of California, San Francisco, San Francisco, CA 94158, USA; 8 Woods Institute for the Environment, Stanford University, Stanford, CA 94305, USA; 9 Division of Epidemiology and Biostatistics, School of Public Health, University of California, Berkeley, Berkeley, CA 94720, USA; 10 Gangarosa Department of Environmental Health, Rollins School of Public Health, Emory University, Atlanta, Georgia, United States; 11 Department of Forestry and Environmental Resources, North Carolina State University, Raleigh, North Carolina, United States

**Keywords:** Sanitation coverage, WASH, fecal contamination, diarrheal disease

## Abstract

**Background:**

Neighborhood-level sanitation coverage may offer significant indirect protection against diarrheal disease, an observation that has been supported by several studies.

**Methods:**

We analyzed the protective effect of neighborhood sanitation coverage using harmonized data from two studies: a randomized control trial (RCT) examining the effectiveness of improved compound-level sanitation and an observational study that collected information on compounds within 100 meters of the RCT study compounds. We developed the Neighborhood Sanitation & Fecal Exposure (NSFE) metric, which estimates the fecal contamination at a study compound based on the demographic and sanitation characteristics of the neighborhood. NSFE is a function of the number of individuals and latrine quality at surrounding compounds, the distance to neighboring compounds, and the effectiveness of hygienic and unhygienic latrines relative to open defecation. We modeled the relationship between NSFE and childhood diarrheal prevalence within RCT study compounds. Finally, we examined counterfactual scenarios to understand how much of the observed diarrheal burden was attributable to neighbors’ latrine quality.

**Results:**

We estimated a 3.6-fold increase in diarrheal prevalence between compounds with the highest and lowest NSFE, with about two-thirds of this association concentrated in compounds between the 90^th^ and 100^th^ percentile of NSFE values. In counterfactual scenarios, we estimate that 15.5% of the entire diarrheal disease burden in children living in the study compounds would be eliminated if all neighboring compounds had high-quality latrines.

**Conclusion:**

Community effects associated with neighborhood sanitation coverage are important drivers of diarrheal disease and should be considered in future designs of sanitation interventions. Our findings support the importance of universal basic sanitation as a target for United Nations Sustainable Development Goal 6.

## Introduction

Diarrhea is a leading cause of dehydration, malnutrition, and mortality in children.^1^ In Bangladesh, like many other low- and middle-income countries, diarrhea-specific mortality has drastically decreased since the 20^th^ century due to widespread interventions, including improved sanitation, handwashing, vitamin supplementation, and oral rehydration therapy.^2^ Despite these reductions, the prevalence of diarrhea remains relatively high among Bangladeshi children (about 5% two-week prevalence in children <5 years in 2022)^3^, indicating that efforts to prevent diarrhea remain insufficient.

Water, sanitation, and hygiene (WASH) interventions have the potential to reduce enteric pathogen transmission and the burden of diarrheal disease,^4^ but individual randomized controlled trials (RCTs) have shown mixed effectiveness. For example, in 2012–2014, individual and combined WASH interventions were tested in parallel RCTs conducted in Bangladesh and Kenya in the WASH-Benefits (WASH-B) study. Neither trial found that WASH interventions impacted child growth, and effects on diarrheal disease were found in Bangladesh only.^5,6^ The WASH-B Bangladesh sanitation, handwashing, and combined WASH interventions (but not water) each reduced the prevalence of diarrhea among children under five by about 2–3 percentage points on the absolute scale.^6–8^ Higher intervention adherence and higher pre-existing access to basic WASH infrastructure in Bangladesh are among the potential explanations for the discrepancy in diarrhea effects across contexts.^5,6,9^

The residual prevalence of diarrhea among intervention households in WASH-B Bangladesh suggests that additional routes of pathogen transmission not closed by the interventions contribute significantly to the burden of diarrhea.^7,10^ WASH-B interventions were household-based, and, on average, only around 10% of households in each geographic cluster received an intervention. Fecal contamination may have originated from the surrounding neighborhood, such as from households with poor or no WASH infrastructure or roaming animals. Meta-analyses of WASH interventions have found high heterogeneity in diarrheal outcomes, including variation by level of community sanitation coverage.^4,11–13^ One systematic review found that sanitation interventions that target an entire neighborhood (rather than a random subset of houses within a community) generally have had higher impact on diarrheal disease.^14^ While the impact of sanitation coverage has long been theorized to have an important role in reducing infectious disease burden beyond the direct impact of access to a household toilet, there is now a growing body of empirical literature supporting the importance of the “the neighborhood effect” of sanitation in pathogen transmission. ^10,15–19^

Pathogens in feces that are not safely contained can travel through the neighborhood environment via animals, person-to-person transmission in the community, and surface water runoff seeping into drinking water.^20^ These mechanisms depend on several characteristics of the area surrounding a household, including population density, availability and quality of latrines in the neighborhood, and the prevalence of open defecation. However, because of challenges around collecting complete data for a whole community, few studies have comprehensively examined the effect of neighborhood sanitation coverage on diarrheal prevalence.^16,21^ Understanding the impact of neighborhood-level sanitation coverage would underscore the importance of the target of achieving universal basic sanitation in line with the Sustainable Development Goal 6. Historically, sanitation interventions have resulted in limited changes to community coverage.^22^ While there is evidence to suggest that sanitation interventions do confer reductions in diarrhea disease,^4^ intervention coverage may be a more important driver of impact than other factors, such as direct sanitation access at the household-level or even the effectiveness of the sanitation intervention to control pathogens along the given pathway. ^8,22^

Studies investigating the association between sanitation coverage and diarrheal risk have typically made several assumptions in their definition of neighborhood sanitation coverage that may obscure the association. First, all neighbors within some radius of a household have equal contributions to the environmental contamination at that household. This assumption does not account for a continuous spatial gradient in which neighbors located closer to a household may contribute more contamination than those farther away. Second, although many studies have used access to a sanitation as a proxy for toilet use,^15,16^ it is likely that open defecation is still practiced occasionally by some household members. ^23 24^

In 2013–2015, an environmental impact study nested within the WASH-B Bangladesh trial^25^ collected neighborhood-level information on sanitation coverage, providing the opportunity to comprehensively examine neighborhood sanitation coverage. A recent analysis of these data^15^ found that the association between sanitation coverage among proximate neighbors (<100 m away from households) and diarrheal outcomes in children was moderate and approached significance (7-day diarrhea prevalence ratio of 0.82 [95% CI: 0.64, 1.04] among those who had 100% sanitation coverage versus less than 100% coverage within 100 meters).

The goal of this present analysis was to create a spatially informed measure of neighborhood sanitation coverage and assess its relationship with diarrheal risk. We first developed a spatially informed neighborhood sanitation metric, the Neighborhood Sanitation & Fecal Exposure (NSFE), which captures the expected amount of contamination a compound experiences. This contamination arises from the demographic (i.e., the number of people residing in each compound) and sanitation (i.e., latrine quality) characteristics of the neighborhood, as well as the distance of each neighboring compound to the study compound. Then, we assessed the association between NSFE and diarrhea, and, finally, we evaluated the potential impact of sanitation interventions on diarrheal prevalence using counterfactual scenarios (i.e., “what would have happened if…?”). that explicitly accounts for neighborhood risks.

## Methods

### Data

We used data from a study conducted throughout four districts of rural Bangladesh: Gazipur, Kishoreganj, Mymensingh, and Tangail; all located in the Dhaka division.^25^ The study enrolled 720 randomly selected compounds that were originally enrolled in the control (n=360 out of 1,362) and sanitation (n=360 out of 696) arms of WASH-Benefits Bangladesh. Throughout this analysis, we refer to these units as “study compounds”. Compounds are multi-family units (usually standalone structures) with a shared courtyard and often a shared latrine. The study compounds were visited eight times over the course of 30 months. At the first visit, investigators collected GPS coordinates, the number of residents, the number of latrines, the observed quality of each latrine. During each visit, a survey was given to ask whether the children under the age of 5 in the compound had diarrhea in the past seven days, and about open defecation practices of household members. Diarrheal disease was defined as passing three or more loose or watery stools or at least one stool with blood. To be consistent with when the information on the neighboring compounds was collected (see below), we use the diarrheal data collected during the second visit in this analysis.

During the second visit, the compounds neighboring each study compound were also surveyed (hereafter referred to as “neighboring compounds”). A neighboring compound was defined as any compound within 100 meters of each of the study compounds. Candidate neighboring compounds were first identified for data collection by the field staff taking 300 steps in any direction from the study compound, and then GPS coordinates were used to remove compounds outside of the 100-meter radius. Like the study compounds, within these neighborhood compounds, information was collected on the number of residents, the number of latrines, and the observed quality of each latrine. In addition, field staff collected information on whether each latrine was hygienic, where it flushed to, and whether feces were observed to be fully contained within a pit or septic tank. Hygienic latrines were defined as an improved facility that did not drain into the environment and where feces were fully contained within the pit or septic tank.^15^ Seven study compounds were missing information on neighboring compounds, leaving 713 study compounds with a total of 1,321 enrolled children under 5 years

### Analytic approach

#### Overview

To address our research goal of creating a spatially informed measure of neighborhood sanitation coverage and assessing its relationship with diarrheal risk, we developed a three-part analysis plan. First, we created a spatially informed neighborhood sanitation metric, Neighborhood Sanitation & Fecal Exposure (NSFE), defined for each study compound. NSFE quantifies the expected amount of contamination arising from the neighborhood. NSFE is a function of: 1) the distance to neighboring compounds, 2) the number of people residing and the presence and quality of latrines at the neighboring compounds, and 3) the expected reduction in contamination arising from the use of hygienic or unhygienic latrines relative to open defecation. We calculated the relative NSFE (rNSFE), as the NSFE normalized by the largest observed NSFE. Second, we used beta–binomial regression to estimate the association between rNSFE and diarrheal prevalence, accounting for potential clustering within compounds. Finally, we applied a series of counterfactual scenarios to this model to estimate how much of the diarrheal burden in the study was attributable to the neighborhood effect and to latrine quality among neighbors, specifically.

#### Neighborhood Sanitation & Fecal Exposure (NSFE)

The NSFE at study compound $C_{i}$ is denoted $\text{NSFE}(C_{i})$. This measure aims to represent the concentration of fecal exposure from all neighbors of compound $C_{i}$, equal to the sum of the fecal exposures $r(C_{j})$ across all compounds $C_{j}$ within 100 meters of compound $C_{i}$. That is, the total exposure is,

$$\text{NSFE}\left(C_{i}\right)=\sum_{j\in \{j:\left|C_{j}-C_{i}\right|\leq 100\}}r(C_{j}).$$

We assumed that fecal exposure from compound $C_{j}$ was greater if the compound was spatially close to study compound $C_{i}$ and if members of the compound practiced open defecation. Thus, we formulated $r(C_{j})$ to account for two main factors: the distance between compounds $C_{i}$ and $C_{j}$ (Factor 1), and the expected fecal exposure from compound $C_{j}$ based on latrine availability and quality (Factor 2). Note that each study compound is included as a neighboring compound of itself (i.e., $i\in \{j:\left|C_{j}-C_{i}\right|\leq 100\}$) to account for exposure within that study compound.

##### Factor 1:

###### Distance to neighboring compounds:

**Figure 1:**
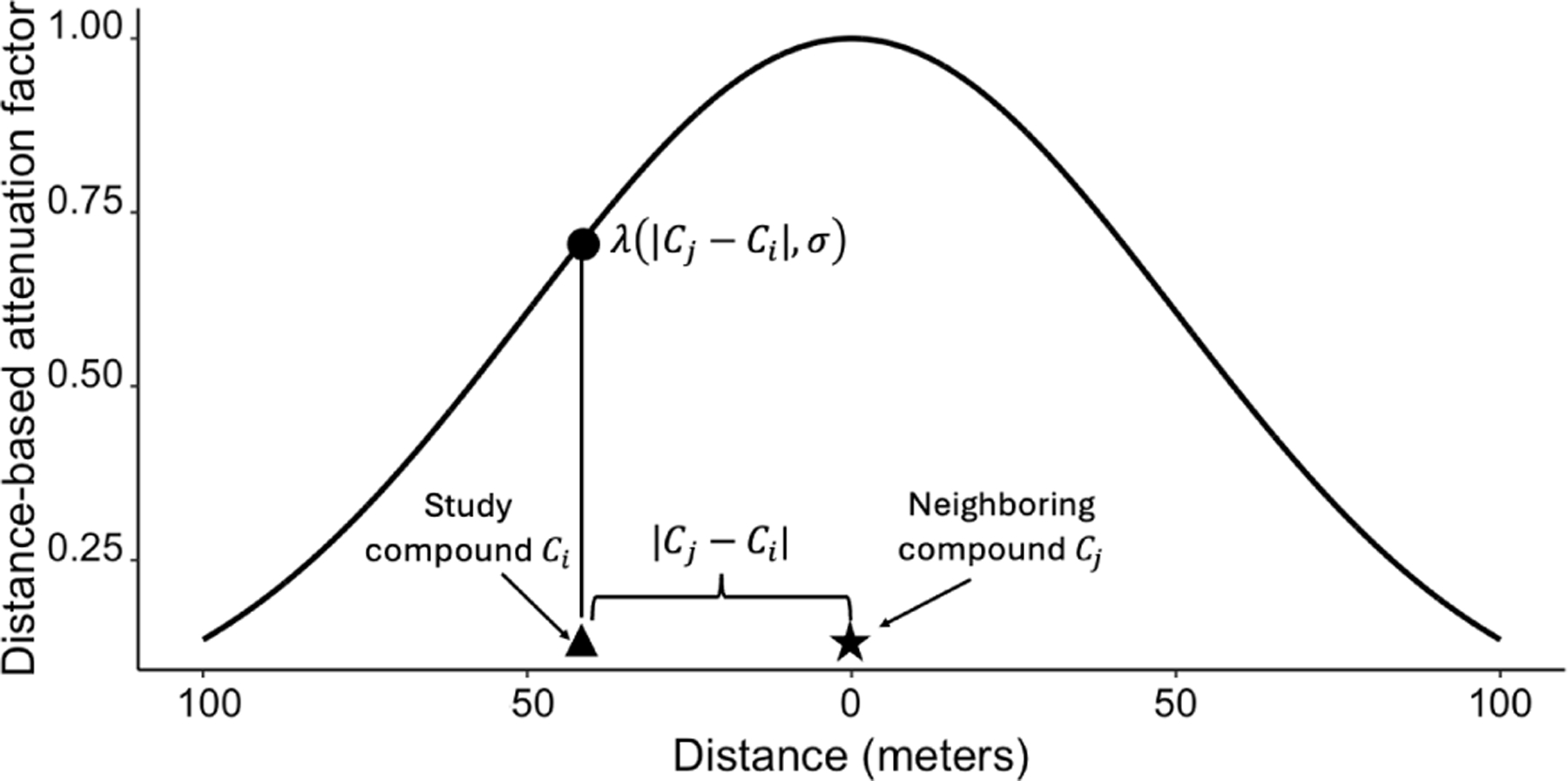
Distance-based attenuation of fecal exposure was assumed to follow a Gaussian distribution, normalized so that the attenuation factor for contamination from compound $C_{j}$ is at its maximum value of 1 at compound $C_{j}$. That is, the fecal contamination originating from neighboring compound $C_{j}$ is multiplied by the value of this normalized Gaussian distribution evaluated at the distance between the two compounds in meters, $\lambda (\left|C_{j}-C_{i}\right|,\sigma )$, where σ is the standard deviation of the distribution.

The first factor incorporates the assumption that the effect of fecal contamination from neighboring compounds diminishes with increasing distance. We modeled this distance-based attenuation of neighborhood fecal exposure using a normalized Gaussian distribution $\lambda (|C_{j}-C_{i}|,\sigma )$, where $\left|C_{j}-C_{i}\right|$ is the distance between compound $C_{j}$ and $C_{i}$ and the highest exposure is to individuals in the same compound ($\left|C_{j}-C_{i}\right|\;=0$ meters away) (Figure 1; Equation S1). The distribution is parameterized by a bandwidth (standard deviation) parameter, σ, which determines the steepness of the fecal exposure drop-off with distance. A higher bandwidth parameter corresponds to a shallower exposure drop-off. Because this parameter cannot be estimated *a priori*, we included it as a sampled parameter (see Parameter Estimation Approach section). An example of how modifying the bandwidth changes the distance-based attenuation of fecal exposure from neighboring compound $C_{j}$ and influences how diarrheal risk is attenuated for compounds at specific distances can be found in Figure S1.

##### Factor 2:

###### Expected fecal exposure from compound $C_{j}$:

This second factor estimates the amount of fecal contamination arising from a neighboring compound as a function of the number of people at a compound, along with the number of latrines and latrine quality (i.e., hygienic or unhygienic). This factor is a weighted sum depending on the relative likelihood of and relative contamination from each of the three types of defecation (open defecation, unhygienic latrine defecation, and hygienic latrine defecation). Specifically, this factor depends on (1) the probability of the type of defecation and (2) the fecal exposure contributed by each type of defecation, scaled by the number of people at compound $C_{j}$:

$$\mu_{j}\left(R_{OD}P\left(OD\right)+RR_{UD}P\left(UD\right)+RR_{HD}P\left(HD\right)\right),$$

where $\mu_{j}$ is the number of residents in compound $C_{j}$, and ${RR}_{UD}$ and ${RR}_{HD}$ are the relative contaminations from defecation in a hygienic and unhygienic latrine relative to open defecation. Since we did not have environmental measurements, we set the contamination amount from open defecation ($R_{OD})$ equal to one. The contamination level of the other latrine types were then estimated as a value relative to open defecation contamination. Because it is challenging to empirically quantify $RR_{HD}$ and $RR_{UD}$ from the literature, we estimated these parameters. A more detailed explanation of our estimation approach is described in the Parameter Sampling Approach section.

These relative contamination factors are multiplied by the probability of each type of defecation in a compound, where $P\left(OD\right)$, $P\left(UD\right)$, and P(HD) are the probability of open defecation, and defecation in unhygienic and hygienic latrines, respectively. While $\mu_{j}$ is available in the data, we did not have information on the sex and age of the members of neighboring compound $C_{j}$. Given defecation behaviors vary between sex and age groups,^23,26^ we estimated the proportion of people in the neighboring compounds that are men, women, and children (in age groups of <3 years, 3–8 years, and 9–15 years), by applying 2022 Bangladesh Census^27^ estimates of the demographic makeup of the population in each of the four districts of Bangladesh (Table S1).

To calculate the probability of each type of defecation, we first calculated the probability of open defecation, $P\left(OD\right)$, by sex and age group using the information we had on open defecation in the study compounds. We constructed a log-binomial regression model, where the outcome was any open defecation (versus none). The predictors in the model were the number of people sharing the latrine, whether the latrine was a flush latrine, and whether the latrine was hygienic; these predictors were selected because they are known to influence open defecation behavior.^23^ The results from this regression separated by demographic group can be found in Table S2. For neighboring compounds with missing model covariates (n=82), we predict the probability of open defecation using the mean of the covariate values for the study compounds in their cluster. Using the coefficients from the log-binomial regression model and the characteristics of the neighboring compounds, we predicted the prevalence of open defecation among the neighboring compounds (Table S3).

The remaining probability mass (i.e., 1−P(OD)) was partitioned into the probability of defecation in either a hygienic or unhygienic latrine (P(UD) and P(HD), respectively). These two probabilities we set to the proportion of hygienic and unhygienic latrines at neighboring compound $C_{j}$. If there were no latrines at the compound, we set $P\left(OD\right)=1$, P(HD)=0, and P(UD)=0.

###### Final model for Neighborhood Sanitation & Fecal Exposure (NSFE):

Multiplying the two factors together, we defined the final equation:

# Eq. 1 $\text{NSFE}\left(C_{i}\right)=\sum_{j\in J:\left|C_{j}-C_{i}\right|\leq 100}\mu_{j}\left(P{\left(OD\right)}_{j}+RR_{UD}P{\left(UD\right)}_{j}+RR_{HD}P{\left(HD\right)}_{j}\right)\lambda_{distance}\left(\sigma ,\left|C_{j}-C_{i}\right|\right)$

**Figure 2:**
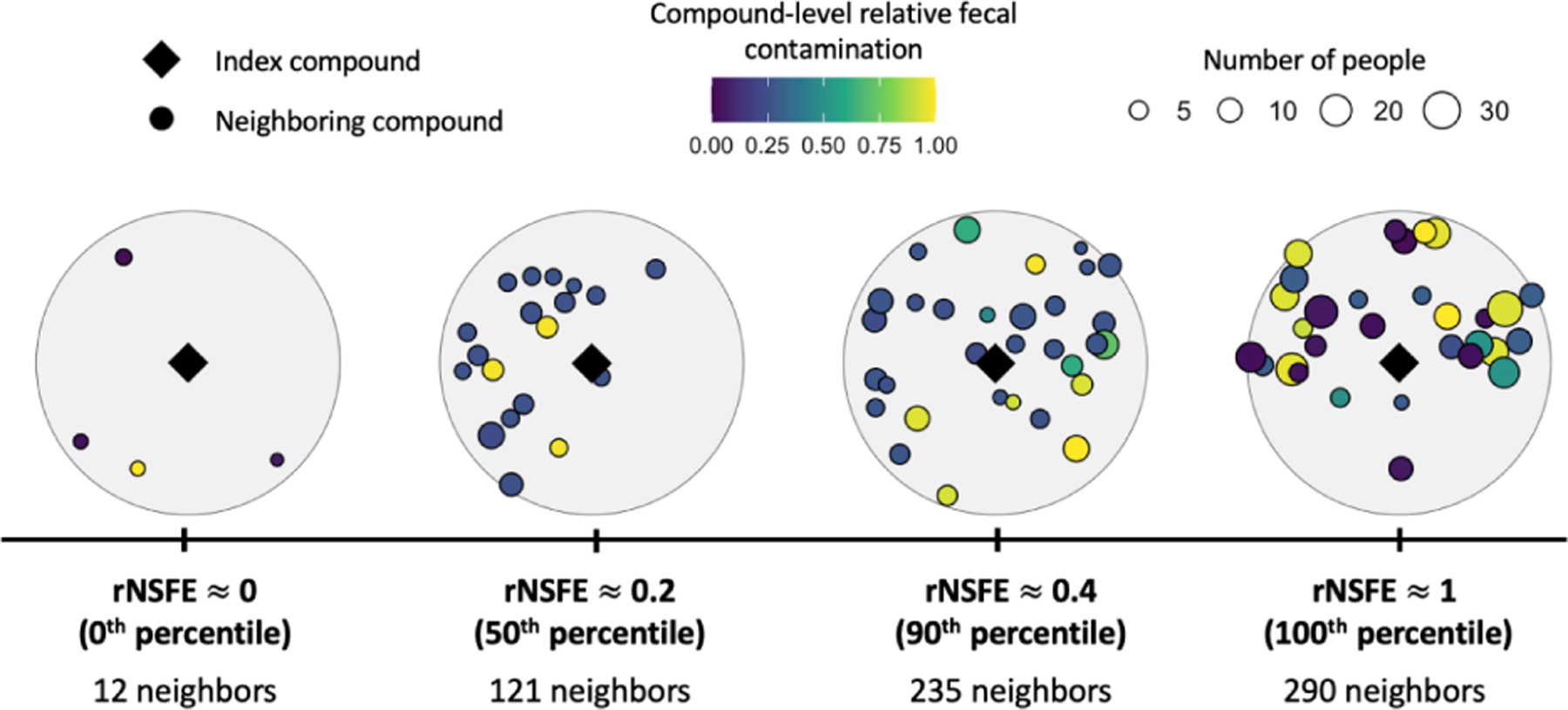
Four study compounds (diamonds) and all neighboring compounds within 100 meters (circles) representative of the relative Neighborhood Sanitation & Fecal Exposure (rNSFE) values of approximately 0 (the lowest possible value), 0.2 (50^th^ percentile), 0.4 (90^th^ percentile), and 1 (the highest possible value). The size of the circles denotes the number of people living in each neighboring compound. Neighboring compounds are colored by the fecal exposure (i.e., contamination level) with yellow representing higher fecal contamination, and dark purple lower.

Table 1 includes all the parameters in the equation and their sources. The full equation, accounting for the five demographic groups we are considering (men, women, children under three years, children between 3–8 years, and children 9–15 years), can be found in Eq. S2. Because NSFE is unitless, we normalized it to the highest observed NSFE to make it more interpretable as a relative measure: $\text{rNSFE}\;=\frac{NSFE}{\max\;\left(NSFE\right)}\;\text{∈}$ [0,1]. An example illustrating the NSFE for four compounds and their neighborhoods can be found in Figure 2.

#### Statistical model

To account for the statistical correlation between children living in the same compound, we modeled diarrheal prevalence using a beta-binomial model, which is a binomial model that allows for overdispersion (i.e., accounting for the fact that the likelihood of diarrhea for children within the same compound is more likely to be similar compared to other compounds with the same levels of the predictors). In the model, the weekly compound-level diarrheal prevalence among children in living the compound ($p_{i})$ is given by:

$$p_{i}=\;\beta_{0}\exp\;(\beta *\text{rNSFE}\left(C_{i}\right)),$$

where the intercept $\beta_{0}$ is the diarrhea prevalence at the compound with the lowest rNSFE (i.e., 0) and exp(β) is the diarrhea prevalence ratio between the compounds with the highest rNSFE (i.e., 1) compared to the lowest.

##### Parameter estimation approach:

To estimate parameters not available in the study data, we use a hybrid estimation–sampling algorithm.^10,28^ In short, we first sampled parameter values for $RR_{UD}$, $RR_{HD}$, and σ from prior distributions (the probability distribution that represents our beliefs about the parameter values before observing any data) and used these values to find the best fitting values of β and $\beta_{0}$ for each set of sampled parameters. We did this 100,000 times. We then calculated the negative log-likelihood (NLL) for each sampled parameter set. Finally we estimated the posterior distributions of the parameters (i.e., the probability distribution of the parameter values given the data) by resampling the parameters 100,000 times using probabilities weighted proportionally to the NLL of each parameter set. The hybrid estimation–sampling algorithm is outlined in more detail in the Supplemental Material. We used K=100,000 sampled parameter sets and N=100,000 resamples, with sampling set $\theta_{s}=\{RR_{UD},RR_{HD},\sigma \}$ and estimated set $\theta_{e}=\left\{\beta ,\;\beta_{0},\varphi \right\}$ where φ is a dispersion parameter that accounts for with-compound clustering (see Supplemental Material). In the estimation step, we minimized the NLL as a function of estimated parameters (given the fixed sampled parameters) using a beta-binomial likelihood.

The priors for sample set $\theta_{s}$ were set using Sobol sampling^29^ with $0<RR_{UD}<2$, $0<RR_{HD}<1$, $RR_{HD}<RR_{UD}$, and 0<σ<200. While we assumed that the relative risk of hygienic latrines ($RR_{HD})$ was less than that of open defecation, we allowed for the relative risk of unhygienic latrines, $RR_{UD}$, to be greater than 1 because, by definition, unhygienic latrines are on the compound property and do not safely contain feces, which may result in higher neighborhood contamination than open defecation, which often occurs off of the compound premises (Table S3).

#### Sensitivity analyses

We conducted two sensitivity analyses. First, to address systematic differences in the compounds in the most densely populated parts of the study area (which generally have the highest NSFE values) we estimated $\beta_{0}$ and β using only the compounds from the bottom 90^th^ percentile of NSFE to determine the extent to which the top 10^th^ percentile of compounds drive the observed associations. Second, we estimated the relationship between NSFE and diarrheal prevalence in two models that did not have the distance-based risk attenuation parameter σ. In other words, these models did not assume that the contamination from compounds was dependent on their distance from the study compound. In the first model, we only consider compounds within 50 meters, and in the second model we consider all the compounds within 100 meters. This model is equivalent to the main model in Eq. 1, without the distance-based attenuation factor (i.e., $NSFE\left(C_{i}\right)=\sum_{j\in J:\left|C_{j}-C_{i}\right|\leq 100\;}\mu_{j}\left(P{\left(OD\right)}_{j}+RR_{UD}P{\left(UD\right)}_{j}+RR_{HD}P{\left(HD\right)}_{j}\right)$). Then, in the hybrid sampling–estimation algorithm we used sampled set $\theta_{s}=\{RR_{UD},RR_{HD}\}$, and the same estimated set as in the main model, $\theta_{e}=\{\beta ,\;\beta_{0},\varphi \}$.

#### Counterfactual scenarios

We estimated two counterfactual scenarios in the study population (i.e., examining “what would have happened if…?”). The first scenario was an intervention where all latrines in the neighborhood were converted to hygienic latrines and the second was if all compounds had the lowest neighborhood risk observed. We defined two counterfactual rNSFE metrics corresponding to each scenario: first, $\text{rNSF}{\text{E}}_{\text{c1}}$ is the neighborhood sanitation and fecal exposure if all neighboring latrines were hygienic (i.e., $\text{rNSF}{\text{E}}_{\text{c1}}(C_{i})=\sum_{j\in J:\left|C_{j}-C_{i}\right|\leq 100}\mu_{j}*RR_{HD}*\lambda_{distance}\left(\sigma ,\left|C_{j}-C_{i}\right|\right))$. Second, $\text{NSF}{\text{E}}_{\text{c2}}$ is the neighborhood sanitation and fecal exposure if all compounds had the lowest possible rNSFE (i.e., 0) which is equivalent to the estimated model intercept, $\beta_{0}$. To calculate the counterfactual diarrhea prevalence for each compound under these two scenarios, we evaluated the best-fit function (based on beta-binomial regression) $\beta_{0}\exp\;\left(\beta *\text{rNSFE}\right)$ at $\text{rNSF}{\text{E}}_{\text{c1}}$ and $\text{rNSF}{\text{E}}_{\text{c2}}$.

Based on these counterfactual scenarios, we decomposed the observed total diarrhea prevalence into three categories: (A) the diarrhea prevalence due to latrine quality in the neighborhood, (B) the diarrhea prevalence due to other neighborhood risks (aside from latrines), and (C) the diarrhea prevalence at the lowest neighborhood risk observed. This decomposition of the prevalence can be visualized as the area between the curves delineating the observed and two counterfactual prevalence lines when the compounds are arranged by rNSFE quantile. From these prevalences, we calculated the following preventable fractions: the preventable fraction due to an intervention that converts all neighboring compounds to exclusively hygienic latrines and the preventable fraction due to a comprehensive neighborhood intervention that eliminates all neighborhood effects. To obtain these values, we divided the respective prevalences by the observed disease burden, i.e., A/(A+B+C) and (A+B)/(A+B+C) for the two preventable fractions, respectively.

## Results

### Descriptive statistics

Across all 713 study compounds included in the analysis, there were an average of 12.5 (IQR: 8–15) neighboring compounds and an average of 115 people (IQR: 70–151; range: 9–425) living within 100 meters of any given study compound. In study compounds, the 7-day prevalence of diarrhea among children under five was 14.2%. We estimate that around two-thirds of defecation across all the neighborhoods occurred in hygienic latrines; however, the range was anywhere between 0–100% depending on the specific neighborhood. Table S3 shows the characteristics of open defecation in the study population broken down by age and sex and the predicted characteristics in the neighbors.

### Neighborhood Sanitation & Fecal Exposure (NSFE) as a measurement of neighborhood sanitation coverage

The first goal of this analysis was to develop the Neighborhood Sanitation & Fecal Exposure metric, designed to quantify sanitation coverage and quality in the neighborhood. Visualizing how neighborhood sanitation coverage is captured by our NSFE metric in the study compounds, Figure 2 shows the neighborhood of four compounds representative of rNSFE values of approximately 0 (lowest possible value), 0.2 (50^th^ percentile), 0.4 (90^th^ percentile), and 1 (highest possible value). NSFE was highly dependent on the population density within 100 meters with compounds with NSFE values in the <5^th^, 45^th^–55^th^, 85–95^th^, and >95^th^ percentile having an average of 29, 114, 198, and 280 neighbors within 100 meters, respectively.

### Association between Neighborhood Sanitation & Fecal Exposure (NSFE) and diarrhea

The second goal of this analysis was to test the association between Neighborhood Sanitation & Fecal Exposure and diarrheal prevalence. The posterior distributions for the model parameters from the beta-binomial regression are given in Table 2. The median posterior estimate for $\beta_{0}$, the diarrhea prevalence at the compound with the lowest NSFE, was 0.10 (i.e., we estimated compounds with the lowest NSFE still had 10% diarrheal prevalence). For the prevalence ratio parameter, exp(β), the median posterior value corresponded to a diarrhea prevalence ratio of 3.6 between the compounds with the highest NSFE compared to the lowest, i.e., compounds with the highest NSFE were estimated to have 36% diarrheal prevalence). The median posteriors values of $RR_{UD}$ and $RR_{HD}$ were 0.9 and 0.4, respectively, meaning that unhygienic latrines were estimated to contaminate 90% as much as open defecation, while hygienic latrines were expected to contaminate 40% as much as open defecation.

**Figure 3:**
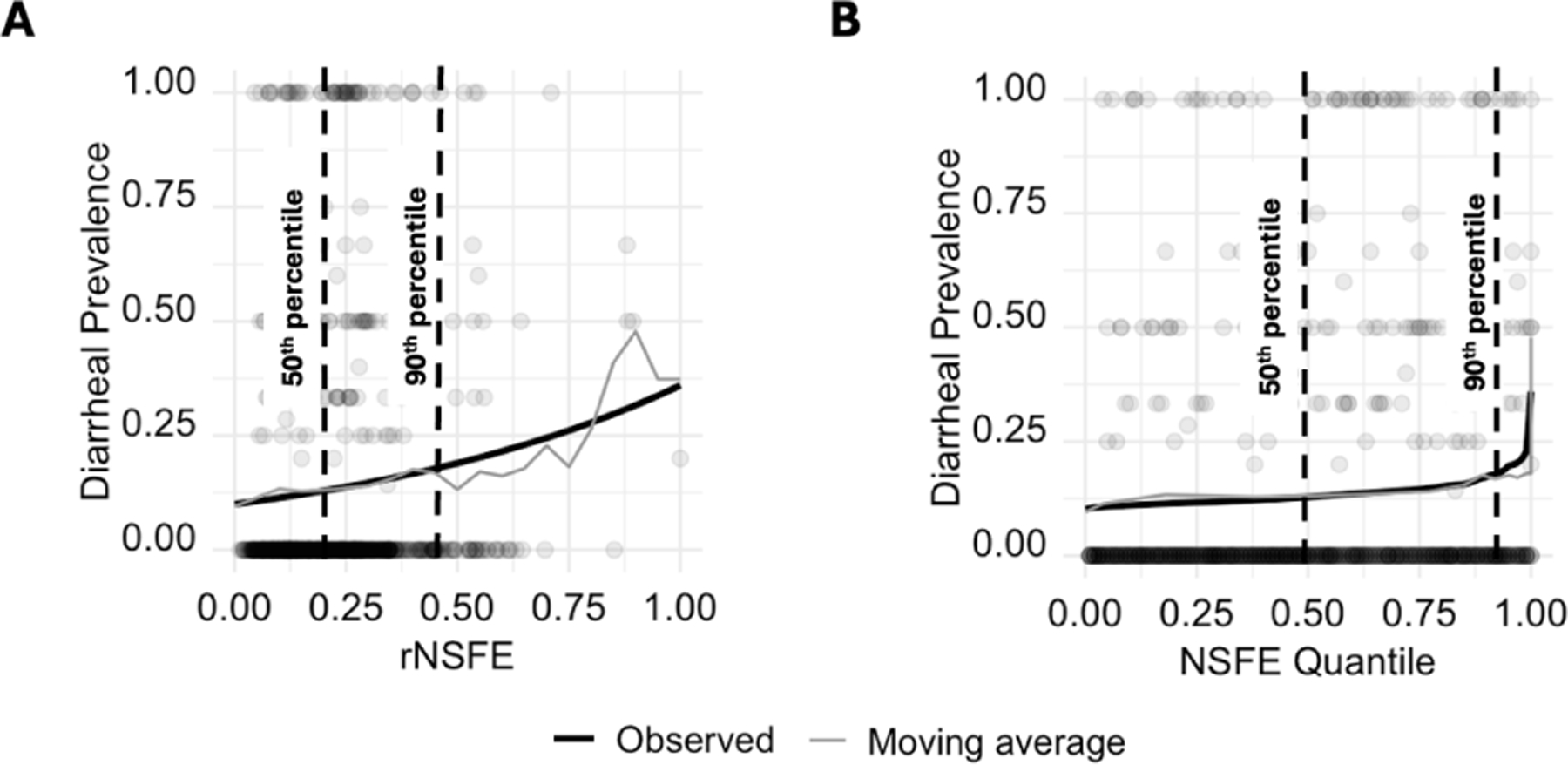
**(A)** The modeled association between the relative Neighborhood Sanitation & Fecal Exposure (rNSFE) and diarrheal prevalence modeled using the median posterior values of $\beta_{0}$ and β (black line) and **(B)** the association from panel **A** transformed by NSFE quantile to account for highly skewed NSFE values across the study compounds. The NSFE value for each compound was calculated using the median posterior estimates for $RR_{HD}$, $RR_{UD}$, and σ, which were 0.9, 0.4, and 127, respectively. The moving average for diarrheal prevalence for children living in study compounds with NSFE of +/− 0.05 (or NSFE quantile +/− 0.05 in **B**) are shown with the grey lines (based on the collected data). The dots show the reported weekly compound-level diarrheal prevalence, with the x-axis value representing the compound’s NSFE and the y-axis value corresponding to diarrhea; prevalence within the last seven days. The more dramatic changes in the moving average for values above NSFE of 0.4 is due to the greater data sparsity in these areas.

Figure 3A shows the association between rNSFE and diarrheal prevalence using the median posterior values of $\beta_{0}$ and β (black line). The rNSFE for each compound was calculated using the median values of $RR_{UD}$, $RR_{HD}$, and σ. The 50^th^ and 90^th^ percentile NSFE values are marked on the figure, highlighting how highly skewed the rNSFE values are across the study. To account for this skew, we plotted the same association, with the x-axis value transformed by quantile (Figure 3B). This version of the figure demonstrates that the majority of increased diarrheal risk is in compounds lying above the 90^th^ percentile of NSFE values, with a shallower association observed in the lower 90% of compounds (the estimated prevalence difference between 0^th^ and 90^th^ percentile was 6 percentage points, compared to 17 percentage points between 90^th^ and 100^th^ percentiles).

The sensitivity analysis where we estimated the values of $\beta_{0}$ and β only including the bottom 90^th^ percentile of NSFE compounds showed very similar results to the main model (see Figure S2). In this model, compounds at the 90^th^ percentile had a diarrheal prevalence of 20%, compared to the estimated prevalence of 19% on the data ran on the full dataset. Both models had intercepts of 10% diarrheal prevalence.

#### Distance-based risk attenuation (bandwidth) parameter:

The posterior distribution of the bandwidth parameter σ had a median value of 127, equivalent to a 30% distance-based risk attenuation at 100 meters and 8% at 50 meters (see Figure S3). To assess the importance of accounting for attenuation in exposure by distance, we conducted a sensitivity analysis to consider the scenario that there is no distance-based attenuation. First, we considered only compounds within 50 meters, and second, we considered all compounds within 100 meters (Figure S4). These two models had similar results to the main model, with an absolute increase in diarrheal risk between the lowest and highest possible rNSFE values of 0.22 and 0.26, respectively (compared to an increase of 0.26 in the main model with distance-based attenuation).

### Counterfactual scenarios

**Figure 4:**
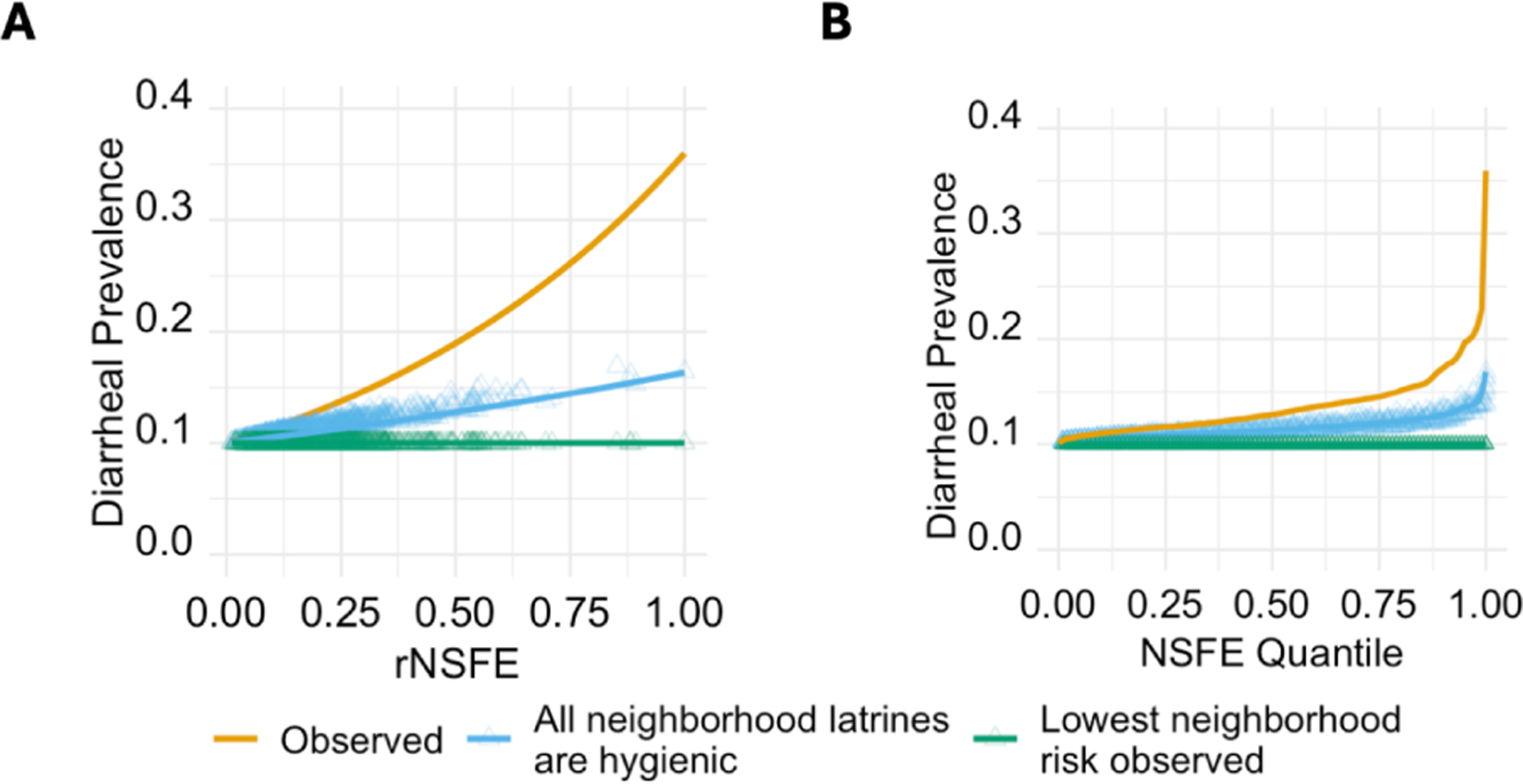
Diarrheal prevalence as a function of the observed association between the relative Neighborhood Sanitation & Fecal Exposure (rNSFE) and diarrheal prevalence (orange), the counterfactual scenario where all neighboring latrines are hygienic (blue), and the counterfactual scenario where all compounds have the lowest neighborhood risk observed (green), **(A)** untransformed and **(B)** transformed by NSFE quantile. Triangles show the counterfactual diarrheal prevalence for individual compounds in the respective counterfactual scenarios; for these points, the x-axis value is the observed NSFE for that compound, and the y-axis value is the original function (orange line) evaluated at the counterfactual NSFE for each compound for each scenario ($\text{rNSF}{\text{E}}_{\text{c1}}$ and $\text{rNSF}{\text{E}}_{\text{c2}}$) relative to the original range of NSFE values.

The third and final goal of this analysis was to test the potential impact of two counterfactual scenarios: where all neighbors use exclusively hygienic latrines and where all compounds have the lowest neighborhood risk observed. In the counterfactual scenario where all neighboring compounds use hygienic latrines (Figure 4), we found a dramatic decrease in diarrheal prevalence in study compounds (blue line; Figure 4). For the highest rNSFE value, the diarrheal disease prevalence dropped from a value of 0.36 to 0.16 for the counterfactual estimate (preventable fraction = 0.56) (Table 3). There was less change in the compounds with lower NSFE values, including, by definition, no change in compounds with a rNSFE of 0.

**Figure 5:**
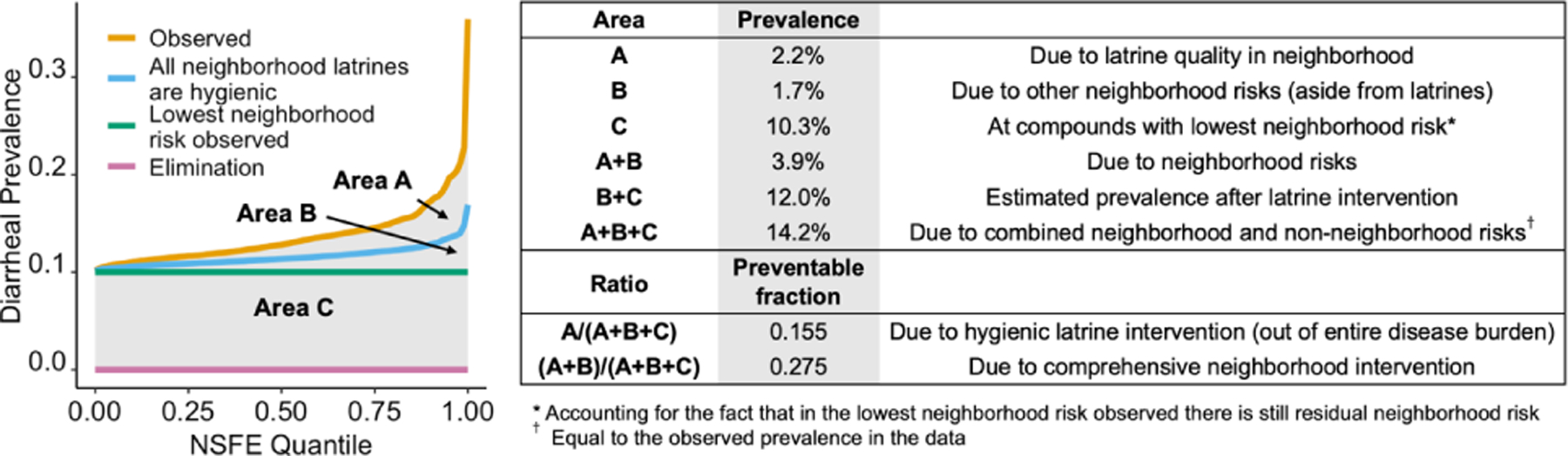
Diarrheal prevalence and preventable fractions under various counterfactual scenarios. In the left figure, the observed association is shown for scale with a zoomed-in version on the right showing the observed association (orange line), counterfactual scenario where all neighborhood latrines are hygienic (blue line), counterfactual scenario where all neighborhoods are equivalent to the lowest neighborhood risk observed (green line), and elimination (pink line). The areas (A, B, and C) between the lines directly translate to the diarrhea prevalence due to (A) latrine quality in the neighborhood, (B) non-latrine associated neighborhood risk, and (C) a counterfactual scenario where all neighborhoods had the lowest neighborhood risk observed. Areas that are proportions of other areas in the graph (e.g., A/(A+B+C)) can be interpreted as preventable fractions.

To better understand the impact of the theoretical counterfactual scenarios, we show the interpretations Figure 5, using the quantile-transformed NSFE. We found that 15.5% of the entire disease burden in children living in the study compounds would be eliminated if all neighboring compounds were switched to hygienic latrines. This results in a prevalence of 12.0% (down from 14.2%). If an intervention were to intervene on all aspects of the neighborhood effect including ones that are unrelated to latrine quality, then 27.5% of the entire disease burden would be eliminated, resulting in a diarrhea prevalence of 10.3% (Figure 5).

## Discussion

The objectives of this analysis were to develop the Neighborhood Sanitation & Fecal Exposure (NSFE) metric, a continuous, spatially informed metric of neighborhood sanitation coverage; quantify the association between NSFE and diarrheal prevalence; and evaluate the potential impact of a neighborhood-level intervention that improves latrine availability and quality. We found a positive association between NSFE and diarrheal prevalence, indicating that low neighborhood sanitation coverage increased diarrheal prevalence. We estimated that 15.5% of the entire childhood diarrheal disease burden in the study population could be eliminated with an intervention that led to exclusive use of hygienic latrines across all the neighborhoods in the study. Our findings agree with previous work^15–19^ that identified neighborhood sanitation coverage as a contributing factor to childhood diarrheal risk and provide further insight into the magnitude of diarrhea prevalence that could be eliminated with a neighborhood-level hygienic latrine intervention.

Understanding the protective effects of neighborhood sanitation coverage on diarrheal outcomes helps elucidate the potential impact of achieving the United Nation’s Sustainable Development Goal (SDG) 6, which includes universal basic sanitation (6.2). Previous work on this topic has identified a positive association between low neighborhood sanitation coverage and diarrhea disease prevalence.^15–19^ However, there is no agreed-upon metric for quantifying neighborhood sanitation coverage, and studies have reported highly heterogeneous effects, ranging from modest and insignificant to large and significant.

Previous work classified neighborhood sanitation coverage into discrete categories such as 0%, partial, or 100% sanitation coverage^15^ and did not account for the specific distances to neighboring compounds, suggesting a need for a more granular approach to exposure classification of neighborhood sanitation coverage. Our NSFE metric was dependent on the characteristics of the neighborhood within 100 meters of a compound including the number of neighbors, the distance to the neighbors’ residences, and the latrine quality and open defecation behavior of the neighbors. The number of neighbors within 100 meters was highly variable in our study area (range: 9–425). As a result, NSFE was both highly driven by and correlated with neighborhood population density. Given the high variation in population density across the study compounds, (ranging from around 10 to 300 people per square kilometer), this approach allowed us to examine NSFE across a wide array of population densities.

We found a substantial association between Neighborhood Sanitation & Fecal Exposure and diarrheal prevalence, namely a 3.6-fold increase in diarrheal prevalence comparing compounds with the highest and lowest NSFE. However, compounds in this Bangladesh site with high NSFE were anomalies—the NSFE values across the study compounds were highly right-skewed. Because very few compounds (less than 10%) had a rNSFE of above 0.4, compounds in the top 90^th^ percentile of NSFE had substantially greater neighborhood risks than other deciles. As a result, when scaling the association by NSFE quantile, the association between NSFE and diarrheal prevalence was highly nonlinear, with about two-thirds of the absolute increase in diarrheal prevalence between the lowest and highest NSFE concentrated in compounds between the 90^th^ and 100^th^ percentile of NSFE values. In other words, for the vast majority of compounds in the study, the association between NSFE and diarrheal prevalence was much more modest.

In the sensitivity analysis where we assessed the impact of removing compounds above the bottom 90^th^ percentile of NSFE, the association remained nearly identical, indicating that the compounds above the 90^th^ percentile did not significantly affect the model fit for these other compounds. Therefore, we do not expect uncontrolled for confounding in the compounds with the highest NSFE scores to affect the interpretation of the results for the compounds in the bottom 90^th^ percentile of NSFE.

In our counterfactual scenario where all the neighboring compounds used exclusively hygienic latrines, we estimated that 15.5% of diarrhea prevalence would be eliminated. Though we estimate 15.5% of diarrhea would be eliminated overall, the preventable fraction was much higher for compounds with higher NSFE (for example, the preventable fraction was 32% for compounds in the 90^th^ percentile of NSFE). This finding indicates that the intervention would disproportionately benefit the most vulnerable compounds. Of the total burden of diarrhea attributed to neighborhood-associated risk (areas A+B, Figure 5), the counterfactual neighborhood-wide latrine intervention would only eliminate about half (56%). The remaining neighborhood-associated risk that is not eliminated with a hygienic latrine intervention (area B, Figure 5) corresponds to aspects of the neighborhood effect that are unrelated to latrine quality. These other pathways might include animals tracking pathogens through the environment or person-to-person transmission in the community.

In agreement with previous work that considered varying distance to neighboring compounds, we found that the distance to neighboring compounds was not an important factor in the neighborhood effect. Contreras et al.,^15^ which used the same data as the present study but a different analytic approach, compared the prevalence of diarrhea in compounds surrounded by compounds with 100% sanitation coverage (defined as at least one latrine per compound) versus <100% coverage for 50 and 100 meters. This study reported similar effect estimates for the two distances considered, both of which showed marginally significant protective effects of 100% sanitation coverage (prevalence ratio of 0.82 [95% CI: 0.64, 1.04] and 0.83 [95% CI: 0.67, 1.02] for analyses considering compounds within 50 and 100 meters, respectively). In our analysis, models that gave higher weights to compounds spatially close to the study compounds in the NSFE calculation (i.e., models with lower values of σ) generally fit the data poorly, indicating that the specific distance to neighboring compounds did not matter as much as overall community-level contamination in explaining diarrheal prevalence. In our sensitivity analysis, the two models that did not incorporate distance-based attenuation (one for only compounds within 50 meters, and one for all compounds within 100 meters) found only modestly different associations between NSFE and diarrheal prevalence compared to the main model (see Figure S4). This finding can be interpreted as suggesting that less distance-based contamination sources, (e.g., person-to-person transmission arising from social interactions in the community) are likely a larger driver of the neighborhood effect than distance-based contamination such as pathogens traveling through runoff. Alternatively, the radius used in this analysis (100 meters) may not provide a large enough scale to truly examine distance-based attenuation of contamination. Other work examining neighborhood-level sanitation coverage have used much larger radii, including defining sanitation coverage on the village-level,^30–34^ and found significant associations between village-level sanitation coverage and diarrheal outcomes, further supporting the hypothesis that relevant transmission pathways are less proximate.

One limitation of our work is that our counterfactual result on the community-wide latrine intervention, which reported the expected reduction in diarrhea from transitioning all neighborhoods to exclusively hygienic latrines, was a function of the median posterior value $RR_{HD}$ (the relative fecal exposure from hygienic latrine defecation versus open defecation). $RR_{HD}$ was informed using the data from this study, and therefore caution should be exercised when extrapolating the results to other settings, especially given that different regions have different hydrogeological features. Finally, we expect there may be some residual confounding in the identified association between NSFE and diarrheal prevalence. The compounds in the most densely populated neighborhoods (which also have the highest NSFE), may be systematically different in ways that are not captured in the NSFE measurement, and not accounting for these differences may have affected the observed association. Our sensitivity analysis was one way to assess the potential for unmeasured confounders, but future work on assessing the potential unmeasured confounders (e.g., socioeconomic status) would provide a deeper understanding of the association between neighborhood sanitation coverage and diarrheal outcomes.

## Conclusion

Understanding the influence of neighborhood-level sanitation on childhood diarrhea risk (the “neighborhood effect”) supports the United Nation’s SDG target 6.2 and the importance universal basic sanitation coverage to achieve maximal health gains beyond what is conferred by individuals within a household that have direct access. Given that the effects of sanitation interventions are primarily indirect,^19^ our work highlights the requirement that sanitation coverage be high in order to achieve the anticipated health gains of sanitation. Explicit models and metrics to capture the neighborhood effect will help provide insight into the mechanisms driving neighborhood level risks.

## Supplementary Material

supplemental

## Figures and Tables

**Table 1:** Parameters included in the Neighborhood Sanitation & Fecal Exposure estimate

Parameter	Source	Symbol
**Compound-specific parameters**		

Number of people at a compound	Data	*μ*_*j*_
Probability of open defecation	Data	P(OD)
Probability of defecation in an unhygienic latrine	Data	P(UD)
Probability of defecation in a hygienic latrine	Data	P(HD)
Distance from study compound (meters)	Data	|$C_{j}-C_{i}|$

**Estimated (sampled) parameters**		

Relative fecal exposure from unhygienic latrine defecation versus open defecation	Estimated	$RR_{UD}$
Relative fecal exposure from hygienic latrine defecation versus open defecation	Estimated	$RR_{HD}$
Distance decay bandwidth parameter	Estimated	σ

**Table 2:** The mean, median, and 95% confidence intervals of the posterior estimates for model parameters: $RR_{UD}$ and $RR_{HD}$ (the contamination of unhygienic and hygienic latrines relative to open defecation, respectively), σ (the distance-based attenuation bandwidth parameter), exp(β), the prevalence ratio between a compound with a rNSFE of 1 and 0, and $\beta_{0}$ the model intercept, or the diarrhea prevalence at the compound with a relative Neighborhood Sanitation & Fecal Exposure (rNSFE) value of 0.

Variable	Mean	Median	95% Confidence interval
exp(β)	3.6	3.6	(2.7, 4.1)
$\beta_{0}$	0.10	0.10	(0.10, 0.11)
$RR_{UD}$	0.8	0.9	(0.2, 1.4)
$RR_{HD}$	0.4	0.3	(0.0, 0.9)
σ	123	127	(43, 196)

**Table 3:** Estimated observed and counterfactual diarrheal prevalence for the two counterfactual scenarios (all neighboring compounds using hygienic latrines and the lowest neighborhood risk observed) for compounds at the 0^th^, 50^th^, 90^th^, and 100^th^ percentile of relative Neighborhood Sanitation & Fecal Exposure (rNSFE) values.

	rNSFE ≈ 0 (0^th^ percentile)	rNSFE ≈ 0.2 (50^th^ percentile)	rNSFE ≈ 0.4 (90^th^ percentile)	rNSFE ≈ 1 (100^th^ percentile)
**Prevalence**				
Observed	0.10	0.13	0.19	0.36
All neighboring compounds using hygienic latrines	0.10	0.11	0.13	0.1
Lowest neighborhood risk observed	0.10	0.10	0.10	0.10

**Preventable fraction**				
All neighboring compounds using hygienic latrines	0.00	0.15	0.32	0.56
Lowest neighborhood risk observed	0.00	0.23	0.47	0.72
