# Supplementary material for: Examining the effect of neighborhood sanitation coverage on childhood diarrheal disease in rural Bangladesh
Source: Int J Hyg Environ Health. Author manuscript; Available in PMC 2026 Jan 23. (PMC12825392; doi:10.1016/j.ijheh.2025.114732)
Supplement: supplemental [file NIHMS2131099-supplement-supplemental.docx]

# Supplemental material

**Equation S1:** Equation for distance-based attenuation of fecal contamination is modeled with a normalized Gaussian distribution where $\left| C_{j}-C_{i} \right|$ denotes the distance between compound $C_{j}$ and $C_{i}$ and $\sigma$ is a bandwidth parameter.

$$\lambda(\sigma,\left| C_{j}-C_{i} \right|)=\frac{\exp\left( -\frac{1}{2}\left( \frac{\left| C_{j}-C_{i} \right|}{\sigma} \right)^{2} \right)}{\sigma\sqrt{2\pi}}.$$

**Figure S1:** **(A)** Distance-based attenuation factor ($\lambda(\sigma,\left| C_{j}-C_{i} \right|))$ as a function of distance from compound $C_{i}$ (denoted $|C_{j}-C_{i}|)$ for four different bandwidth ($\sigma$) values, $\sigma=10$ (yellow), $\sigma=50$ (orange), $\sigma=100$ (pink), and $\sigma=200$ (purple). **(B)** The relationship between the value of $\sigma$ and the distance-based attenuation factor for compounds that are at a distance of 25, 50, and 100 meters away from compound $C_{i}$ are denoted with the dashed, dot-dash, and solid lines, respectively. In both panels, relationships are plotted for compounds $C_{j}$ of varying distance from compound $C_{i}$ and indicated by shapes: 25 m (squares), 50 m (circles), and 100 m (triangles). Both panels demonstrate that increasing $\sigma$results in the same neighboring compound $C_{j}$ contributing a higher fecal exposure to compound $C_{i}$ (i.e., lower distance-based attenuation of fecal exposure). Effectively, this means that pathogens originating from compound $C_{j}$ have a higher influence on the risk of diarrhea at compound $C_{i}$ in our model.


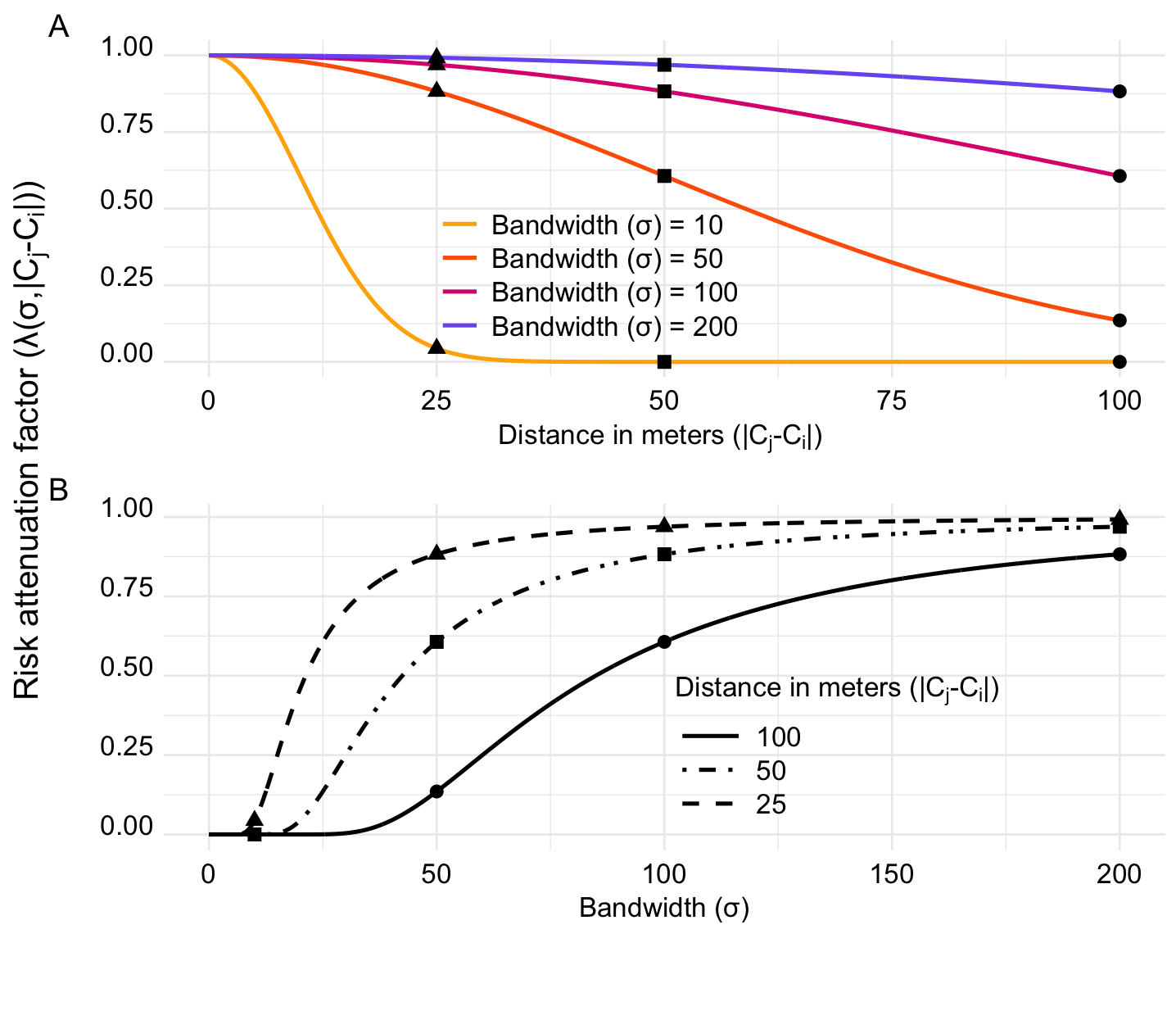


**Table S1:** Bangladesh district population sizes for the four districts in the analysis, by age group and sex (2022)^1^

|  | Men | Women | Children  $\boldsymbol{\geq}$ 8 < 15 | Children $\boldsymbol{\geq}$ 3 < 8 | Children <3 | Total |
| --- | --- | --- | --- | --- | --- | --- |
| Gazipur | 876135 **(37%)** | 813281 **(34%)** | 221396 **(9.1%)** | 245025 **(10%)** | 210501 **(8.9%)** | 2366338 |
| Kishoreganj | 674849 **(28%)** | 745611 **(31%)** | 297355 **(12%)** | 385084 **(16%)** | 319978 **(13%)** | 2422877 |
| Mymensingh | 1269619 **(29%)** | 1355474 **(31%)** | 509545 **(12%)** | 648244 **(15%)** | 529252 **(12%)** | 4312134 |
| Tangail | 967212 **(32%)** | 1088239 **(36%)** | 331086 **(11%)** | 373353 **(12%)** | 301408 **(10%)** | 3061298 |

**Table S2:** Prevalence ratios and 95% confidence intervals of open defecation for different demographics

|  | Open defecation prevalence ratios (95% confidence) | | | | |
| --- | --- | --- | --- | --- | --- |
|  | Men | Women | Children  $\geq$ 8 < 15 | Children $\geq$ 3 < 8 | Children <3 |
| Intercept | 0.20  (0.16,0.25) | 0.04  (0.02, 0.06) | 0.18 (0.12,0.26) | 0.81 (0.75,0.88) | 0.96 (0.95,0.98) |
| Number of people sharing latrine | 1.00 (0.97,1.02) | 0.98  (0.93, 1.04) | 0.95 (0.91,0.99) | 0.97 (0.96,0.98) | 1.00 (1.00,1.00) |
| Flush latrine (y/n) | **0.42* (0.36,0.49)** | **0.61***  **(0.43, 0.87)** | **0.43* (0.34,0.55)** | **0.75* (0.71,0.80)** | **0.93* (0.92,0.94)** |
| Hygienic latrine (y/n) | **0.52* (0.44,0.61)** | **0.52***  **(0.36, 0.75)** | 0.80 (0.61,1.03) | **0.87* (0.82,0.93)** | 0.99 (0.97,1.00) |

*indicates significance at a level of $\alpha=0.05$.

**Table S3:** Characteristics of open defecation in the study population and predicted in the neighbors. Prevalence of open defecation in the neighbors was estimated using a regression model trained on the study compound data (see **Table S2**). The predicted prevalence of open defecation was higher in the neighboring compounds than the study compounds because none of the neighboring compounds received the sanitation intervention.

| WASH-B participants  (n=63,879) | Men  (n=15,873) | Women  (n=16,593) | Children 8-15 years  (n=7,989) | Children 3-8 years  (n=9,277) | Children under 3 years  (n=14,147) |
| --- | --- | --- | --- | --- | --- |
| Open defecation, ever, n (%) | 855 (5.4) | 307 (1.9) | 380 (4.8) | 3,449 (37.2) | 11,816 (83.5) |
| Open defecation occurs at the compound, n (%) | 196 (22.9) | 183 (59.6) | 160 (42.1) | 2,825 (81.9) | 9,499 (80.4) |
| Open defecation occurs in the village, n (%) | 98 (10.2) | 17 (5.5) | 29 (7.6) | 27 (0.8) | 6 (0.1) |
| Open defecation frequency (others in the compound) |  |  |  |  |  |
| Daily, n (%) | 320 (2.7) | 233 (1.9) | — | — | — |
| Occasionally, n (%) | 308 (2.6) | 123 (1.0) | — | — | — |
| Never, n (%) | 11,070 (94.6) | 11,805 (97.1) | — | — | — |
| Travel time to open defecation area, mean in minutes (SD) | 1.1 (3.0) | 0.3 (0.8) | 0.4 (1.5) | 1.0 (2.0) | 0.9 (1.0) |
| Neighbors (predicted) |  |  |  |  |  |
| Open defecation (%) | 11.5 | 5.3 | 10.1 | 50.4 | 91.9 |

**Equation S2:** total neighborhood fecal exposure formula, accounting for demographic groups

$$NSFE\left( C_{i} \right)=\sum_{j\in J:\left| C_{j}-C_{i} \right|\leq100} \begin{aligned} [\mu_{m,j}\left( R_{OD}P\left( OD \right)_{m,j}+RR_{UD}P\left( UD \right)_{m,j}+RR_{HD}P\left( HD \right)_{m,j} \right)+ \\ \mu_{w,j}\left( R_{OD}P\left( OD \right)_{w,j}+RR_{UD}P\left( UD \right)_{w,j}+RR_{HD}P\left( HD \right)_{w,j} \right)+ \\ \mu_{c09,j}\left( R_{OD}P\left( OD \right)_{co9,j}+RR_{UD}P\left( UD \right)_{co9,j}+RR_{HD}P\left( HD \right)_{co9,j} \right)+ \\ \mu_{c59,j}\left( R_{OD}P\left( O \right)_{c59,j}+RR_{UD}P\left( UD \right)_{c59,j}+RR_{HD}P\left( HD \right)_{c59,j} \right)+ \\ \mu_{cu5,j}\left( R_{OD}P\left( OD \right)_{cu5,j}+RR_{UD}P\left( UD \right)_{cu5,j}+RR_{HD}P\left( HD \right)_{cu5,j} \right)]\cdot\lambda(\left| C_{i}-C_{j} \right|) \end{aligned}$$

Where:

- $\mu_{m,j}, \mu_{w,j}, \mu_{co9,j}, \mu_{c59,j}, \mu_{cu5,j}$: number of men, women, and children between the ages of 8-14, 3-7, and under 3, respectively in compound $j$.
- $P\left( OD \right)_{m,j}, P\left( OD \right)_{w,j}, P\left( OD \right)_{co9,j}, P\left( OD \right)_{c59,j}, P\left( OD \right)_{cu5,j}$: probability of **open defecation** among men, women, and children between the ages of 8-14, 3-7, and under 3, respectively in compound $j$.
- $P\left( UD \right)_{m,j}, P\left( UD \right)_{w,j}, P\left( UD \right)_{co9,j}, P\left( UD \right)_{c59,j}, P\left( UD \right)_{cu5,j}$: probability of **defecation in an unhygienic latrine** among men, women, and children between the ages of 8-14, 3-7, and under 3, respectively in compound $j$.
- $P\left( HD \right)_{m,j}, P\left( HD \right)_{w,j}, P\left( HD \right)_{co9,j}, P\left( HD \right)_{c59,j}, P\left( HD \right)_{cu5,j}$: probability of **defecation in an unhygienic latrine** among men, women, and children between the ages of 8-14, 3-7, and under 3, respectively in compound $j$.

### Hybrid sampling-estimation to obtain values of$,$ $RR_{UD}$, $RR_{HD}$,${\sigma, \beta}_{0}$, $\beta$ and $\varphi$

**Input:** $K:$ number of samples; $N:$ number of resampled values where $N\leq K$; sampling set: $\theta_{s}=\{RR_{UD},RR_{HD},\sigma\}$; estimated set: $\theta_{e}=\{\beta_{0},\beta,\varphi\}$; information about all neighboring compounds ($C_{i})$to $C_{i}$: ($P\left( OD \right)_{j},P\left( UD \right)_{j},P\left( HD \right)_{j},$and distance between them $\left| C_{i}-C_{j} \right|$.

1. Generate $Kx3$matrix$\theta_{s}^{K}$ using a Sobol sample where $K$ is the number of samples desired.
2. **for** $\theta_{s}^{k}$ in 1:$K$ (i.e., for each of the $K$ parameter sets) **do**:
3. Run beta-binomial regression (here, we used the glmmTMB function in R) to find the values of $\beta_{0}$, $\beta,$ and $\varphi$ that minimize the beta-binomial negative log-likelihood (NLL) given by:

$$\log\left( \mathcal{L}\left( \beta_{0},\beta,\varphi\right) \right)=\sum_{i=1}^{n} \log\binom{N_{i}}{Y_{i}}+\log\left( B\left( Y_{i}+\alpha_{1,i}, N_{i}-Y_{i}+\alpha_{2,i} \right) \right)-log(B\left( \alpha_{1,i},\alpha_{2,i} \right))$$

Where $N_{i}$ is the number of children in compound $i$, $Y_{i}$ is the number of diarrhea cases in compound $i$, $B\left( x,y \right)=\frac{\Gamma(x)\Gamma(y)}{\Gamma(x+y)}$ is the beta function, and $\alpha_{1,i}$ and $\alpha_{2,i}$ are the compound-specific parameters of the beta distribution given by:

$$\frac{\alpha_{1,i}}{\alpha_{1,i}+\alpha_{2,i}}=p_{i}^{k}=\beta_{0}exp(\beta\sum_{j\in J:\left| C_{j}-C_{i} \right|\leq100} \mu_{j}\left( P\left( OD \right)_{j}+RR_{UD}^{k}P\left( UD \right)_{j}+RR_{HD}^{k}P\left( HD \right)_{j} \right)\lambda\left( \left| C_{i}-C_{j} \right|,\sigma^{k} \right))$$

$$\frac{1}{\alpha_{1,i}+\alpha_{2,i}}=\varphi$$

where $p_{i}^{k}$ is the compound-level diarrheal prevalence, $P\left( OD \right)_{j}, P\left( UD \right)_{j},{P\left( HD \right)}_{j}$ are the pre-determined probabilities of each defecation type for neighboring compound $j$, $RR_{UD}^{k}, RR_{HD}^{k}$ and $\sigma^{k}$ are in $\theta_{s}^{k}$, and $\beta_{0}$, $\beta$, and $\varphi$ are the estimated parameters in $\theta_{e}$. To directly maximize the likelihood as a function of the estimated parameters $\{\beta_{0},\beta,\varphi\}$, the above equations can be rearranged as follows:

$$\alpha_{1,i}=\frac{p_{i}^{k}}{\varphi} \alpha_{2,i}=\frac{1-p_{i}^{k}}{\varphi}$$

1. Define $\theta_{e}^{k}$ as the *estimated* parameter set that achieved the minimum negative-log likelihood (NLL) given $\theta_{s}^{k}$, then set $\theta^{k}=\{\theta_{s}^{k},\theta_{e}^{k}\}$. Save $\theta^{k}$ and corresponding minimum NLL $\mathcal{L}\left( \theta^{k} \right)$ in some output matrix*.*

**end**

1. Once you have $\mathcal{L}\left( \theta^{k} \right)$ and $\theta_{e}^{k}$ for all $k\in K$, define two sets of weights for $\theta^{k}$: normalized and unnormalized weights:

*Unnormalized weights:* $w^{k}\mathcal{=L}\left( \theta^{k} \right)K$, where $\mathcal{L}\left( \theta^{k} \right)$ is the minimum NLL in step 2 (i.e., the NLL corresponding to $\theta^{k}$).

*Normalized weights:* $\bar{w}^{k}=w^{k}/(\sum_{k\in K} w^{k})$ or $\bar{w}^{k}=\frac{\exp\left( \log\mathcal{L}\left( \theta^{k} \right)+\psi\right)}{\sum_{k\in K} exp(\log\mathcal{L}\left( \theta^{k} \right)+\psi)}$

1. Sample $N$ parameter sets with replacement from $\{\theta^{k}\}$ with the probability of selection determined by the normalized weights $\bar{w}^{k}$.

**Output:** $N$ parameter sets corresponding to t the resampled distribution of $\theta$.

**Figure S2**: Sensitivity analysis only including compounds below the 90^th^ percentile of NSFE (blue) and model using full data (black). The diarrheal prevalence in each compound is shown in the blue dots. The results show very similar associations, with a slightly steeper association between NSFE and diarrheal prevalence. In this model, compounds at the 90^th^ percentile had a diarrheal prevalence of 20%, compared to the estimated prevalence of 19% on the data ran on the full dataset. The intercepts were the same between the two models.


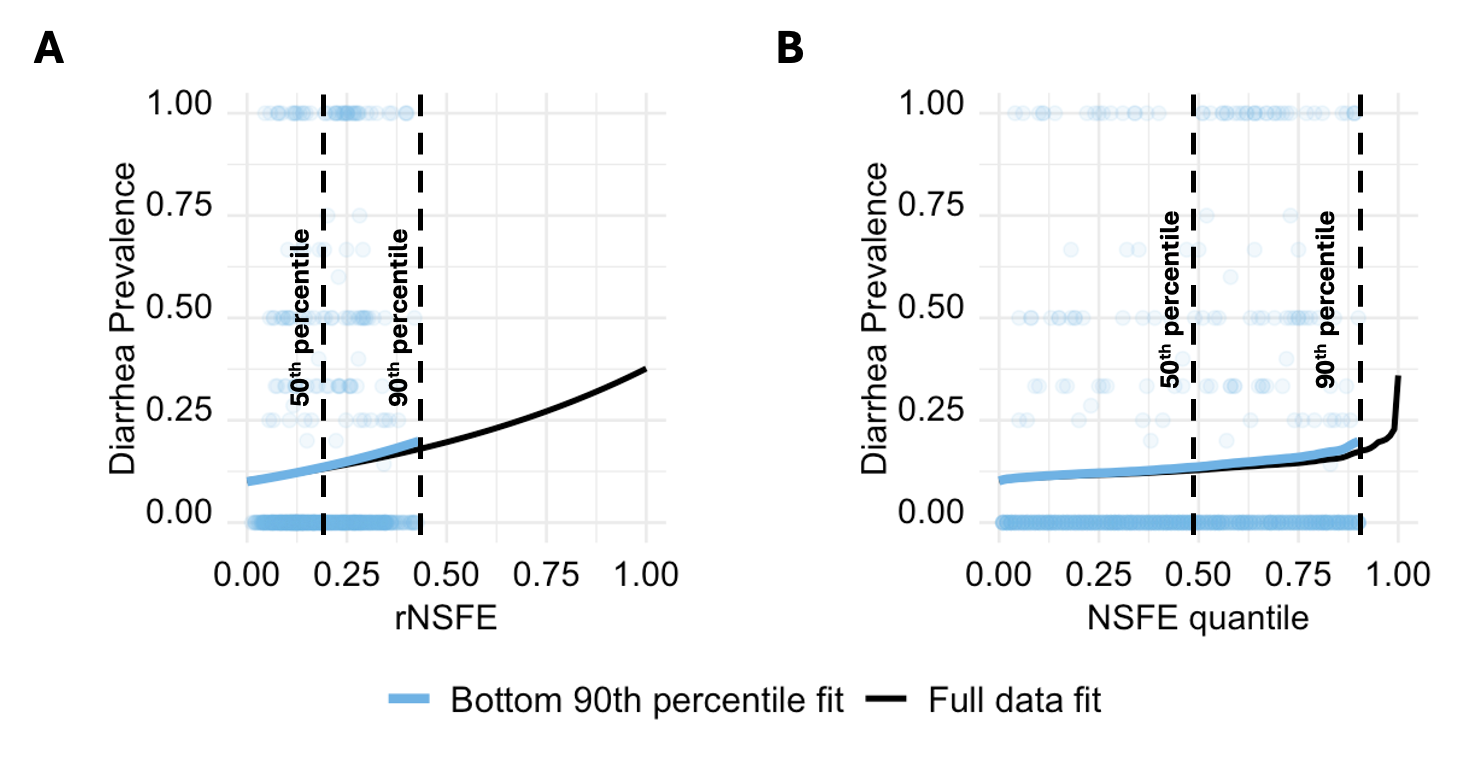


**Figure S3**: Posterior distribution of distance-based attenuation parameter, $\sigma$ (left), and corresponding distribution of attenuation of risk at 50 meters (middle) and 100 meters (right). These plots show that the posterior distribution of the distance-based attenuation parameter is high, corresponding to low values of risk attenuation at 50 meters (mode=4%) and 100 meters (mode = 15%).


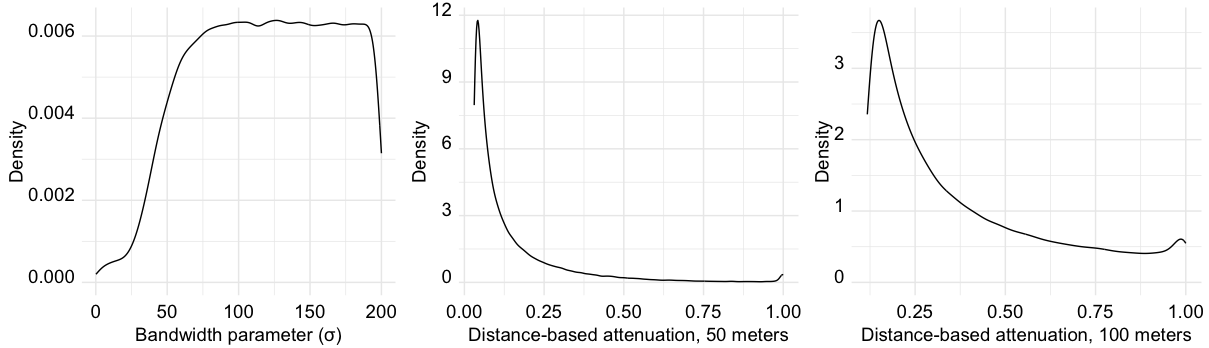


**Figure S4:** Diarrheal prevalence versus relative NSFE (left) and by relative NSFE quantile (right) for three models: two with no distance-based attenuation considering only compounds within 50 meters (blue), and all compounds within 100 meters (yellow), and the third model considering all compounds within 100 meters with a distance-based attenuation parameter (pink). For each model, the intercept ($\beta_{0}$) and slope ($\beta$) parameters for each model were obtained from the median posterior values based on a beta-binomial likelihood. The graph with the x-axis transformed by the quantile of NSFE score (right) was included due to highly skewed NSFE scores across the compounds.


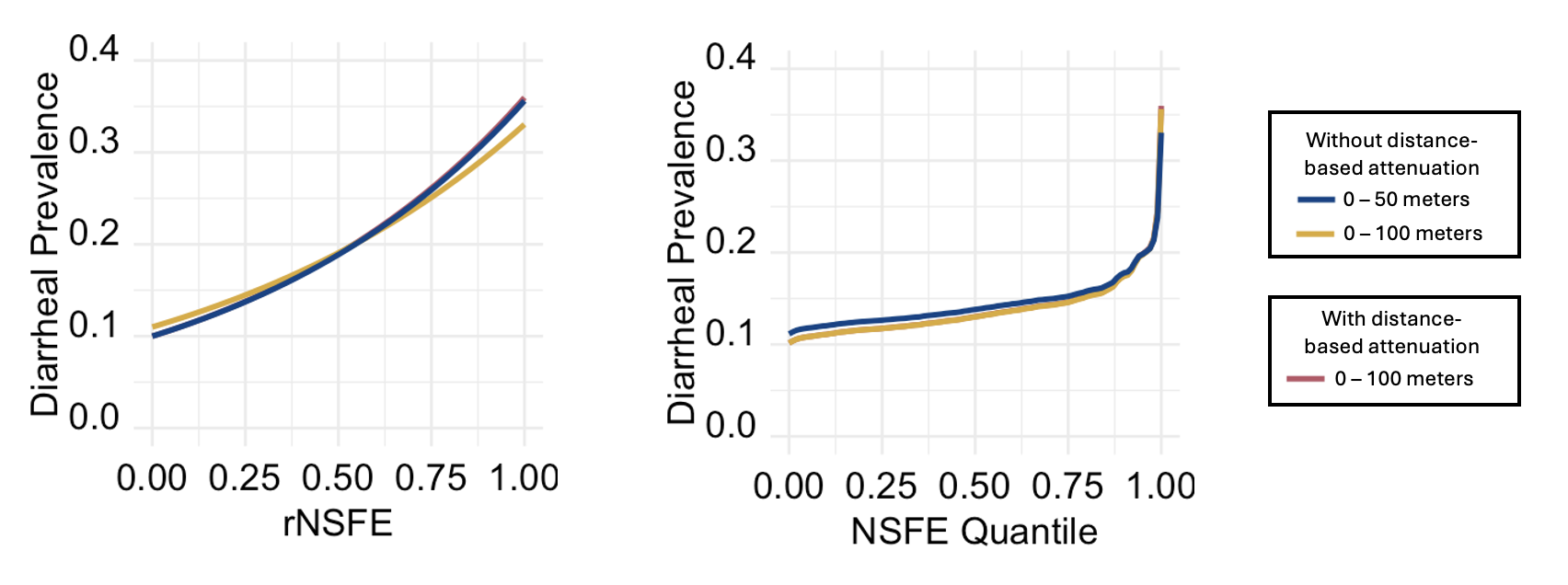


**References**

1. Population-and-Housing-Census - বাংলাদেশ পরিসংখ্যান ব্যুরো-গণপ্রজাতন্ত্রী বাংলাদেশ সরকার. Accessed September 25, 2024. https://bbs.gov.bd/site/page/47856ad0-7e1c-4aab-bd78-892733bc06eb/Population-and-Housing-Census
